# Cholecystitis, with Special Reference to Etiology and Diagnosis

**Published:** 1907-12

**Authors:** T. E. Potter

**Affiliations:** St. Joseph. Mo.


### CHOLECYSTITIS, WITH SPECIAL REFERENCE TO ETIOLOGY AND DIAGNOSIS.

BY T. E. POTTER, M. D., ST. JOSEPH, MO.

---

Within the past two decades, but few, if any regions of the human body have been so well studied, scientifically, as that of the epigastric and right hypochondriac, which is largely occupied by the liver and its belongings. In this study, the gall bladder has been found the source of much pathology in its immediate neighborhood, as well as remote troubles of a less subjective nature, and it has received more attention than perhaps any other portion.

It has been found to be the seat of frequent inflammations both acute and chronic, as a sequelae of such inflammations, empyema, contraction, hydrocholeo-cystitis, stones, stenosis of the ducts, hypertrophy of the liver and adhesions to the loops of the bowels and omentum, malignancy, and remote troubles like general prostration, dyspepsia, angina pectoris, etc.

Assuming these statements to be correct, then it certainly behooves us as physicians and surgeons to look well into the causes of the inflammations, which if understood well, will enable us not only in the beginning to bring about speedy resolution, but in many instances to prevent unpleasant sequelae; if they should occur, an early understanding of them and their source makes it much easier to find a remedy.

Believing as we do, that all inflammations are the result of bacterial infection, we do not hesitate to assume that cholecystitis is, no matter what form it takes, a bacterial disease. Not only is the inflammation of the bladder caused by micro-organisms, but the gall stones so often found are formed as a result of their work. In the inflammed gall bladder a great many pathogenic bacteria, principally the coli communis, typhoid, pneumococci, and pyogenic organisms have been found. A question of much interest is "How can bacteria get into the gall bladder and do much damage in a fluid that is considered aseptic?" The home of the bacillus coli communis is in the alimentary canal; in typhoid fever, the typhoid bacilli are found here in connection with the duodenal and Peyer's glands. Pyogenic microbes are found with dysentery and in pus, wherever found in connection with the intestines. The pneumococci may enter the general circulation from the lung, or the sputum may be swallowed and the microbes carried, as other microbes are, to the liver through the portal circulation.

We know, too, that the gall bladder is connected with the alimentary canal by the ductus communis choledochus and the cystic duct; but infection is not likely to pass up these tracts, as they are constantly filled or have passing through them the biliary fluid, which is sterile and washes them away. The infection could not get to the gall bladder through adhesions with the alimentary tract, as this would have to exist first in the gall bladder before the adhesions. The most rational conclusion as to the course of infection is through the portal circulation, whose blood is collected from the alimentary canal and mesentery and then distributed to the liver. In this blood are found the infectious bacteria, which are separated from it by the liver cells, and pass with the bile into the gall bladder. It will hold pathogenic bacteria for a while without allowing them to reproduce themselves; but if the bile has these germs in it and they are permitted to remain for any length of time, they will multiply. So we find by studying this subject, that persons who lead a sedentary life, or fast a great deal, are much more liable to have cholecystitis, and especially cholelithiasis. Persons whom we suspect of having a predisposition to stones should not be allowed to take long fasts, on the other hand they should eat between meals, so as to keep digestion going on all the time and the viscus empty. The gall bladder only acts as a reservoir for bile to be used in digestion.

I have inquired closely into the past history in the cases that have passed under my observation, and find that a large majority of them have had typhoid fever. The history, may in some, run back several years.

In an article written by Dr. Camac, a former interne in the Johns-Hopkins Hospital, (American Journal of Medical Sciences 1899.) he said, "Chiari found the typhoid baccilli in twenty out of twenty-three cases." There is no doubt that these bacilli are the most frequent pathogenic bacteria found, and the microscopical examination of the composition of gall stones shows them in the center of these bodies.

These opinions are only a few out of many as to the source of gall stones. This theory may at first be doubted, and it may be difficult to understand how stones could possibly come from this source. A Dr. Smith (Buffalo Medical Journal, January 1891) gives a good solution of the question. He says, "when local conditions, or the character of the bacteria, lead to an infection of the catarrhal inflammatory type in the bile tracts, suppuration does not take place, but the deposit of cholestrin and bile salts about a focus of bacterial activity leads to gall stone formation. The colon bacilli and typhoid bacilli have lately been found by numerous observers as the basis of biliary calculi."

After reviewing this subject we come to the following conclusions: That the most probable cause of infection of the gall bladder is not through the intestinal walls, nor through the hepatic tissue, nor through the duodenum through the hepatic ducts, but through the blood of the portal system; and from this source we not only have the catarrhal and parenchymatous inflammations, with occasional empyema, but sometimes malignancy caused by the presence of stones.

We now desire to study the diagnosis of cholecystitis. The symptoms which direct us to a proper conclusion are not all present in any one case, and different forms and stages give us different symptoms. Prominent objective points are not always present, and we may have to arrive at a diagnosis by observing minor and subjective signs. Among the most prominent symptoms is tenderness immediately under the ninth intercostal cartilage. This, in my judgment, is as valuable a symptom as tenderness in the region of McBurney's point is, of appendicitis. The tenderness may extend down for two or three inches. To elicit it, press the fingers up under the ribs, and require the patient to take a deep inspiration. He will complain of pain, and in many instances, if the fingers are firmly held, the inspiration is instantly stopped, as though there had been a sudden attack of pleurisy. There may be some swelling or fullness over the region of the gall bladder, and percussion gives a general sense of uneasiness. By placing the patient on his hands and knees, the gall bladder may occasionally be felt to drop forward, and it can be outlined, especially if it contains stones. The stomach is usually deranged, and the patient not only suffers from indigestion, but severe gastralgia, that can only be relieved by emesis. I never have a patient with these symptoms, but that I at once suspect cholecystitis and stones. There may be slight jaundice, frequently none, yet the close observer will notice that there are few cases that do not at sometime or other show some slight tinging of the conjunctiva. There may be chills and fever, sometimes two rigors a day attended with high fever, which only lasts for a short time and then disappears. These chills may not return for several days, when they occur as before, and are often diagnosed as chronic malaria.

The patient is often of a morose disposition, or suffers with great depression at times. Sometimes there is emaciation and again there is irregularity in the heart beat, as well as great excitement of this organ. I had a patient once who was so nervous and prostrated by this that he could not walk up an ordinary flight of stairs without great exhaustion and when he stopped he would tremble all over, and his heart beat from 120 to 130 per minute, yet there was no disease of this organ. He had never had colic or jaundice, and when I told him what was the matter, he would not listen to me until jaundice set in two weeks later, when he permitted me to operate, and recovered. Angina pectoris is sometimes a symptom and can only be relieved by this operation.

Hepatic colic will aid in forcing a conclusion as to the presence of stones. Still there may be some doubt until they are found in the discharges. These stones are usually found after an attack of colic, and if they have facets upon them, they show that they have had companions to rub against. After the passage of stones through the duct, the tenderness is usually increased. Occasionally the gall bladder becomes very much distended, and will reach as low as Poupart's ligament. It may be mistaken for an apendicular abscess, and only an exploratory incision or aspiration enables us to determine which is present.

I have noticed in several cases where the gall bladder was full of stones, or where there was empyema or hydrocholecystitis, that the administration of calomel and other active cholagogues increased the pain in the region of the liver, and this pain was often attended with general tenderness over the organ. I can only account for such pain in this way; there being no bladder for the residue of bile, the ducts become overdistended and a condition of cholangitis is excited.

As to the treatment, we will not enter into the discussion of the subject extensively. It is divided into both medical and surgical, but I believe when the acute inflammation has subsided, and a chronic condition is established, that but little can be accomplished by medicinal treatment, and an operation is justifiable whether there is evidence of stones or not. I think a man may live much longer with stones in the gall bladder, than he can with many other conditions, and the surgeon is not required to take generally as active measures when there are stones, as when there is empyema, hydrocholecystitis, adhesions, or a general catarrhal condition. To operate and establish drainage is certainly the thing to do. It allows the liver, through the gall bladder, to evacuate itself well without effort. It allows the thickened and chronically inflamed ducts with their lining, to get well without effort. In fact it puts these ducts at rest and at the same time allows this excretory material to be cast off and by doing this prevents autointoxication. I have seen a jaundiced skin clear up in two days after making ventral fixation and opening the bladder at the fundus, and the patients appetite improve from the first. I have frequently seen the hypochondriac, morose and melancholic conditions get better so fast that the patient would be greatly encouraged from the beginning.

As to the use of medicine to correct chronic cholecystitis and to prevent the formation of stones, I think there is little benefit in it. I have thought, after using olive oil thoroughly for weeks and months, that it was of decided benefit. It does certainly relieve the attacks, although its constant use becomes very nauseating. The question is, "How can olive oil be of service?" Stuart gives the following explanation: "With unobstructed pancreatic flow it is not improbable that by virtue of the fat, splitting ferment, steapsin, a portion of the oil is disassociated in the duodenum into its components, fatty acid and glyceryl, and nascent glycerin is introduced into the rectum, withdrawing water, causing hyperemia and inducing irritation of the different nerves of the part with which it comes in contact, and thus leading to active reflex peristalsis."

The sodium phosphate and other agents have been advised without limit, but I do not believe we can hope to get any substantial benefit short of the knife, and the simplicity of this operation made early, is within reach of any physician who has anything like a surgical mind. It is true, if the bladder has to be removed, or there is great contraction of the same, or if there are stones in the duct, it requires many times great surgical skill. There may not be much inflammation in some of the cases and the bladder may have resumed to some extent its normal condition, but upon opening the abdomen and making an examination, adhesions will be found between the gall bladder, omentum tranverse colon and sometimes to the duodenum. So it would be foolishness on the part of the internal medicine man to hope to cure his patient when these conditions exist without resorting to the knife.

